# Computational Design of Enantiocomplementary Epoxide Hydrolases for Asymmetric Synthesis of Aliphatic and Aromatic Diols

**DOI:** 10.1002/cbic.201900726

**Published:** 2020-03-05

**Authors:** Hesam Arabnejad, Elvira Bombino, Dana I. Colpa, Peter A. Jekel, Milos Trajkovic, Hein J. Wijma, Dick B. Janssen

**Affiliations:** ^1^ Biotransformation and Biocatalysis, Groningen Biomolecular Sciences and Biotechnology Institute University of Groningen Nijenborgh 4 9747 AG Groningen The Netherlands

**Keywords:** computational design, enantioselectivity, epoxide hydrolase, molecular dynamics, stilbene oxide

## Abstract

The use of enzymes in preparative biocatalysis often requires tailoring enzyme selectivity by protein engineering. Herein we explore the use of computational library design and molecular dynamics simulations to create variants of limonene epoxide hydrolase that produce enantiomeric diols from *meso*‐epoxides. Three substrates of different sizes were targeted: *cis*‐2,3‐butene oxide, cyclopentene oxide, and *cis*‐stilbene oxide. Most of the 28 designs tested were active and showed the predicted enantioselectivity. Excellent enantioselectivities were obtained for the bulky substrate *cis*‐stilbene oxide, and enantiocomplementary mutants produced (*S*,*S*)‐ and (*R*,*R*)‐stilbene diol with >97 % enantiomeric excess. An (*R*,*R*)‐selective mutant was used to prepare (*R*,*R*)‐stilbene diol with high enantiopurity (98 % conversion into diol, >99 % *ee*). Some variants displayed higher catalytic rates (*k*_cat_) than the original enzyme, but in most cases *K*_M_ values increased as well. The results demonstrate the feasibility of computational design and screening to engineer enantioselective epoxide hydrolase variants with very limited laboratory screening.

## Graphical abstract

**Overcoming bottlenecks**: This paper describes the use of computational design to obtain limonene epoxide hydrolase variants that show regioselective attack of water and thereby form enantiomeric diols from *meso*‐epoxides. Most of the 28 designs that were examined in the laboratory carried multiple mutations, retained stability, and gave diols with the predicted chirality.

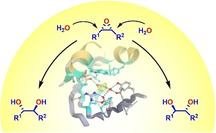

## Introduction

The use of biocatalysis in chemistry is an attractive option for many synthetic processes, especially for preparing fine chemicals and bioactive compounds.^1^, ^2^, ^3^, ^4^, ^5^, ^6^, ^7^ Although nature provides an enormous diversity of industrially useful enzymes, they often must be engineered to meet industrial process requirements.^8^, ^9^ In case of pharmaceutical synthesis, of special importance are chemoselectivity, compatibility with harsh reaction conditions and product enantiopurity.^1^, ^10^ Consequently, extensive studies have been carried out on controlling and improving enzyme selectivity by protein engineering, often through directed evolution,^11^ which led to enzymes with improved selectivity in kinetic resolution of enantiomers and better performance in asymmetric transformation of prochiral compounds.^8^, ^10^, ^12^

Whereas directed evolution is very successful, it requires high‐throughput screening methods. In case of enzyme enantioselectivity, screening is possible by chiral chromatography or by the use of quasi enantiomers in NMR or MS^13^ but this may be time‐consuming and expensive. Directed evolution becomes complicated when no high‐throughput expression is available, such as in case of enzymes that must be produced in fungi. Several methods have been proposed to overcome these bottlenecks, such as optimizing strategies for library construction, for example, by focusing mutations in different combinations around the active site,^14^, ^15^, ^16^ and by incorporating structural^9^ or phylogenetic information.^17^, ^18^ Another option is the use of computational tools to design improved enzymes.^19^, ^20^, ^21^, ^22^, ^23^ Statistical methods have also been used.^24^, ^25^ Biophysics‐based computational protocols have emerged as powerful platforms for the engineering of thermostable and organic‐solvent compatible enzyme variants.^26^, ^27^, ^28^, ^29^, ^30^, ^31^ Multiple mutations can be explored simultaneously, allowing for larger jumps in sequence space than directed evolution and structure‐based rational mutagenesis. Furthermore, in silico screening of enzyme variants by docking and high‐throughput molecular dynamics simulations makes it possible to predict enzyme properties and decrease library size for experimental evaluation from thousands to dozens.^32^, ^33^

In this study, we explore a computational framework (catalytic selectivity by computational design, CASCO)^32^ for obtaining enantiocomplementary epoxide hydrolases. This framework uses the Rosetta scoring function and search algorithm^19^, ^34^ to generate libraries of primary designs. Next, high‐throughput molecular dynamics (MD) simulations with scoring the frequency of occurrence of reactive (or near‐attack) conformations (NACs)^35^, ^36^, ^37^ are used for ranking and to select a small set of variants that qualify for laboratory testing. The frequency of reactive poses during MD simulations can explain the selectivity of computationally designed enzyme variants.^38^, ^39^, ^40^ For that purpose, generating multiple MD trajectories with independent assignment of initial atom velocities gives much better sampling of accessible conformational space than the use of a single trajectory running over a long simulation time.^41^, ^42^, ^43^, ^44^, ^45^ Accordingly, to enable screening of thousands of Rosetta designs by MD, CASCO uses 20–80 of such short MD simulations for scoring conformational stability of designed reactive enzyme–substrate complexes. The MD step thus examines if the conformation of the enzyme substrate complex, which is partially constrained during the Rosetta design step (NAC), will be maintained in short MD runs, or whether that reactive conformation is immediately lost, for example by movement of the substrate to a non‐reactive pose. We used this approach earlier to predict enantioselectivity in kinetic resolutions catalyzed by haloalkane dehalogenases.^46^

The potential of this computational approach was illustrated for limonene epoxide hydrolase (LEH) redesign in a previous study^32^ where we observed that the performance of the best enzymes in a 37‐variant library obtained by the CASCO framework was similar to that of the best variants obtained by screening approximately 4700 variants generated by the CASTing strategy for directed evolution.^47^ Nevertheless, computational library design for enzyme engineering has serious challenges, of which reliability of predictions is an important example. To further explore the possibilities and limitations of computational redesign, we designed and examined a novel set of enantioselective limonene epoxide hydrolase (LEH) variants.

**Scheme 1:**
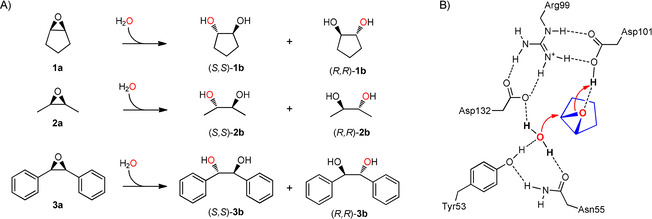
Conversion of epoxides by limonene epoxide hydrolase. A) regioselective hydrolysis of *meso*‐epoxides examined in this study. B) Catalytic mechanism of LEH, illustrated with *proRR* hydrolysis of cyclopentene oxide.

LEH catalyzes the hydrolysis of epoxides by activating a bound water molecule for nucleophilic attack directly on one of the substrate's oxirane carbons.^48^ The water is positioned by H‐bonds to Asn55 and Tyr53 while Asp132 abstracts a proton from the water, which enables nucleophilic attack as a hydroxy ion (Scheme 1). At the same time, Asp101 protonates the epoxide oxygen, which makes it a better leaving group. The reaction is concerted.^49^ In case of *meso*‐epoxides, the stereochemical outcome is determined by regioselectivity of the attack and the products are enantiomers. LEH has been extensively used as a model system for exploring the use of directed evolution strategies to engineer enantioselectivity, and many variants have been described.^47^, ^50^, ^51^, ^52^, ^53^, ^54^, ^55^ We previously examined the use of computational design and screening to improve stability and control enantioselectivity.^26^, ^29^, ^32^

In this work, we investigated three small sets of enantiocomplementary epoxide hydrolase variants for converting *meso*‐epoxides (*cis*‐2,3‐butene oxide, cyclopentene oxide, and *cis*‐stilbene oxide) to their corresponding (*R*,*R*)‐ or (*S*,*S*)‐diols. For each target enantiomer, five top‐ranked new variants were experimentally characterized to explore how challenging it is to position each of the substrates uniquely in the active site. We also measured *k*_cat_ and *K*_M_ values for selected variants. This showed that the *k*_cat_ of some variants was higher than of the thermostable LEH‐P variant, from which all mutants were derived. However, the Michaelis constants (*K*_M_) were almost always higher as well, which decreases catalytic efficiency. The results confirmed the working hypothesis that obtaining unique binding orientations, reflected in high enantioselectivity, was easier with the bulkier substrates. Mutants converted *cis*‐stilbene oxide to diols with an enantiomeric excess (*ee*) of >99 %. Small‐scale preparative reactions were carried out.

## Results

### Computational design

**Figure 1:**
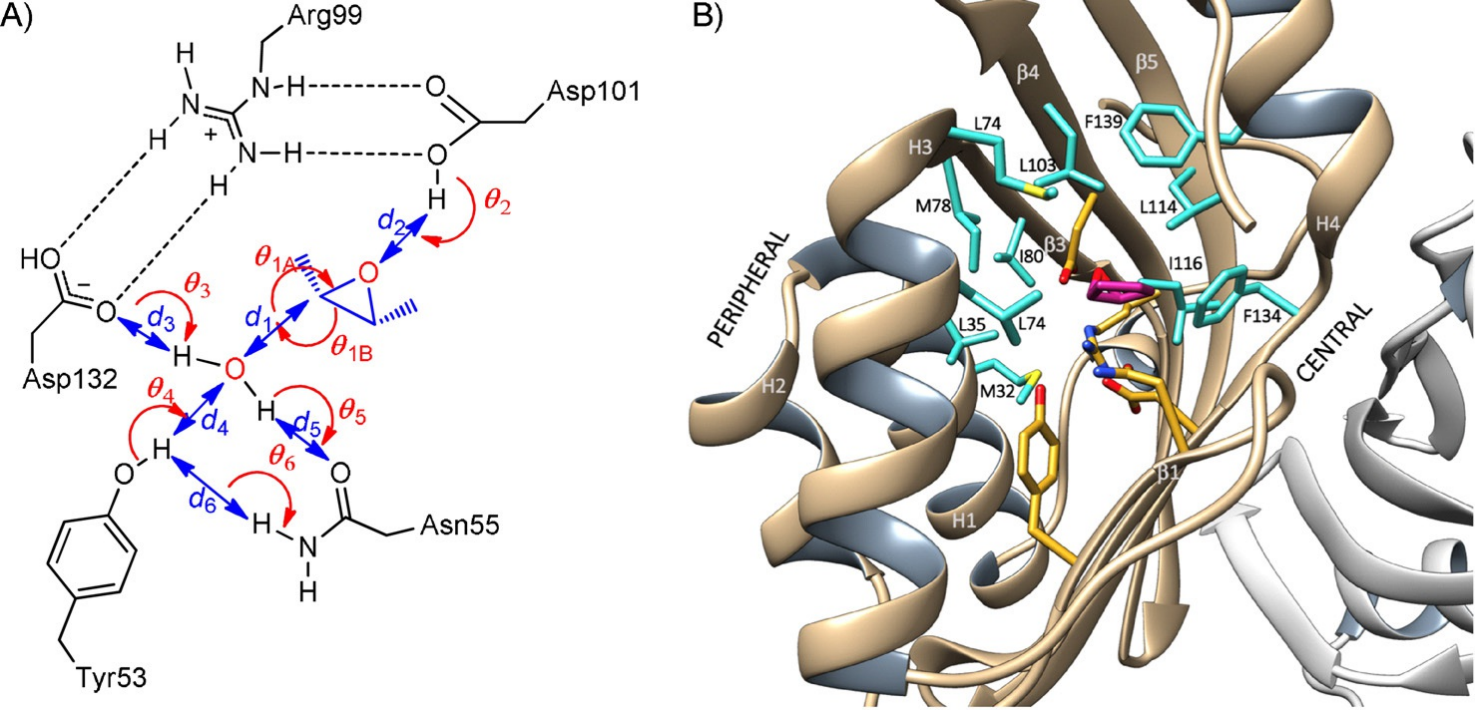
Design of limonene epoxide hydrolases for asymmetric conversion of *meso*‐epoxides. A) NAC criteria used to predict the enantioselectivity of diol formation from 2,3‐butene oxide by MD simulations. The same criteria were used for all three epoxides. Angles and distances are defined as followed: for the nucleophilic attack angle θ_1A_=128–163°, θ_1B_=128–163° and *d*_1_=0–3.22 Å. For the H‐bonds θ_2‐6_=120–180° and *d*_2‐6_=0–3.50 Å. B) selection of target positions (cyan) in PDB structure PDB 4R9K. Catalytic residues are shown in yellow, and the substrate in magenta. The targeted amino acid positions are either located in the peripheral structural elements H1 (M32, L35), H3 (L74), and β3 (M78, I80, V83) which border the *proRR* side of the substrate binding pocket; or in the central region, which forms the *proSS* side of the binding pocket and consists of H4 (F139), β4 (L103), β5 (L114, I116) and β6 (F134). The secondary structure elements are defined as follows: N‐loop (residues 1 to 23), H1 (24 to 35), H2 (40 to 46), H3 (64 to 75), H4 (135 to 143), β1 (52 to 56), β2 (60 to 62), β3 (79 to 91), β4 (94 to 105), β5 (111 to 123) and β6 (126 to 133).

The enantioselectivity of limonene epoxide hydrolase is dependent on the regioselectivity of water attack within the active site. To design epoxide hydrolase variants for enantioselective conversion of the three substrates (**1 a, 2 a, 3 a**, Scheme 1), we performed a series of design calculations for these substrates. Substrates were docked in the active site and placed in a reactive configuration using restraints. This included a hydrogen bond between the leaving oxygen and D101, a close distance between the nucleophilic water and the attacked oxirane carbon, and close to linear orientation of the nucleophilic water, the oxirane carbon, and the epoxide oxygen. Next, the Monte Carlo search algorithm of Rosetta was used to optimize the identity and side chain geometries of amino acids surrounding the active site for either *proRR* or *proSS* attack of the nucleophilic water on the epoxide carbon (Scheme 1). The reactive geometries were essentially defined as a near attack conformation (Figure 1 A). The explored sequence space was created by targeting 11 selected positions around the active site (Figure 1 B) with randomization to any of the nine hydrophobic residues (AFGILMPVW). This way, Rosetta generated variants forming a low energy complex with the target substrate either in the *proRR* or *proSS* orientation, resulting in thousands of possible *proRR* and *proSS* designs per substrate (Table 1). These sets of primary Rosetta designs represent mutant libraries enriched in the desired phenotype.

**Table 1:** Molecular dynamics screening of designed enzyme variants.

CASCO step	Criteria	No. of designs remaining for substrate		
		**1 a**	**2 a**	**3 a**
Total Rosetta designs		28 795	33 252	20 714
Rosetta designs with a unique sequence		4125	4732	6232
Designs remaining after MD screening				
5×10 ps	*ee* ^pred^ >97 %	711	990	2504
10×10 ps	*ee* ^pred^ >97 %	524	723	2205
20×10 ps	*ee* ^pred^ >97 %	378	587	1631
40×10 ps	*ee* ^pred^ >98 %	170	314	1284
80×10 ps	*ee* ^pred^ >98 % + [NAC]^pref^ >5 %	142	283	978^[a]^
5×100 ps	*ee* ^pred^ >98 %	63	181	442^[b]^
Rosetta designs passing all criteria		1.5 %	3.8 %	7.1 %

[a] [NAC]^pref^ indicates the NAC frequency for the preferred product enantiomer. Additional criterion [NAC]^pref^ >10 %. [b] Additional criterion [NAC]^pref^ >5 %.

To computationally screen these libraries in an orthogonal manner by the likelihood of showing the correct regioselectivity of water attack, we performed molecular dynamics simulations. From MD trajectories, enantioselectivities were predicted by scoring the fraction of time that the enzyme–substrate complex is in the same reactive *proRR* or *proSS* conformation used during the Rosetta design step (near‐attack conformations, NACs, Figure 1 A). Instead of a single or a few long MD simulations, we performed a large number of parallel MD simulations with independently assigned initial atom velocities (HTMI‐MD), because this gives more extensive conformational sampling and better agreement with experimental results than single long simulations.^41^, ^42^, ^43^, ^44^, ^45^, ^46^ For each of the 15 000 Rosetta designs, at least five independently initialized MD runs of 10 ps were performed (Table 1). Designs that passed initial selection rounds were subjected to more MD simulations, up to 80×10 ps and 5×100 ps. NACs were counted on the fly and their frequency was averaged for each design. The ratio between averaged NAC frequencies for *proRR* and *proSS* attack conformations was used as a predictor for enantioselectivity using Equation (1). This MD screening of the primary libraries decreased the number of designs by a factor of 15 to 70 (Table 1).

It was noticed during in silico screening by MD that a larger fraction of the stilbene oxide designs displayed high NAC frequencies and high predicted enantioselectivities than what was found with cyclopentene and butene oxide designs. As a result, more stilbene oxide designs survived the final selection (7.1 %) than designs for cyclopentene oxide **1 a** (1.5 %) and butene oxide **2 a** (3.8 %), even though the selection criteria were set significantly more strict for stilbene oxide than for the other substrates (Table 1). This suggests a better occupancy of reactive orientations for stilbene oxide, and more restricted conformations of the stilbene oxide designs than of the designs with the two smaller substrates, at least during MD simulations.

Visual inspection of the top‐ranked designs was carried out to verify that there were no noticeable structural problems that would decrease catalytic activity or enantioselectivity. For every target enantiomer, the variants predicted to have a high *ee* ^pred^ for that product were ranked, those with the highest [NAC]^pref^ first, and variants were inspected until five variants were identified for each target enantiomer. It was noticed that for cyclopentene oxide **1 a** and butene oxide **2 a** there were few designs with obvious errors. In 25 inspected designs for those two substrates there were only five with recognizable structural errors (one was unusually flexible, three had such a spacious active site that reorientation of the substrate seemed likely, and in one mutant there was a new H‐bond to the epoxide oxygen, Table S1). In contrast, 10 of the 20 inspected variants for stilbene oxide displayed structural problems. Four designs appeared too spacious (Supporting Information) and six designs had a water positioned such that attack on the unintended carbon atom of the epoxide seemed likely, even though the NAC analysis did not suggest this.

### Experimental characterization

The top five designs for each product enantiomer of the three substrates were investigated experimentally. Two designs (26A, 45A) were selected twice, both for *proRR* hydrolysis of cyclopentene oxide **1 a** and butene oxide **2 a** (Table 2). We also included three designs predicted to have *proRR* selectivity by molecular dynamics simulation while they were originally designed using Rosetta to have *proSS* selectivity. The 28 new LEH variants were constructed in the thermostable LEH‐P template described earlier^56^ by sequential rounds of QuikChange mutagenesis. The use of a thermostable parent enzyme increases the chance that protein function is maintained upon introduction of mutations that may be too destabilizing in a mesostable template and is also reported for directed evolution protocols.^57^, ^58^, ^59^ The LEH template used here (LEH‐P, *T*_m,app_=70 °C, PDB 4R9K) is not the most stable enzyme from our previous work, as it lacks the disulfide bonds of the most stable variant (*T*_m,app_=85 °C, PDB 4R9L).^56^

**Table 2:** Predicted and observed activities of computationally redesigned limonene epoxide hydrolase variants.

Enzyme		Mutations^[a]^	Computational			Experimental		
			NAC %	NAC %	*ee*_pred_	*ee*_exp_	Act.	*T*_m,app_
			proRR	proSS	[%]	[%]^[b]^	[%]^[c]^	[°C]
		cyclopentene oxide (**1 a**)						
LEH‐P						14	0.12 U mg^−1^	70
Designs for (*R*,*R*)‐1 b	1A	M32L_M78W_I80V_L103F_F139W	5.63	0.000	100	32	2	63.5
	43A	L74I_L103V_F134Y_F139W	3.86	0.040	98	9	29	67.0
	59A	M78L_L103V_L114G_I116F_F139I	2.59	0.008	99	6	1	67.0
	45A	M32L_L74I_L103V_L114W_I116L_F134G_F139W	2.44	0.000	100	–^[d]^	<1	–
	46C	M32L_L103V_L114A_I116F_F139W	2.08	0.000	100	46	24	57.0
Designs for (*S*,*S*)‐1 b	3A	M32L_L35W_I80A_I116V_F139W	0.280	36.5	−98	−74	41	–
	4C	M32L_L35W_I80G_V83I_I116V_F139W	0.208	24.1	−98	−80	55	48.5
	24A	M32A_M78I_I80F_L103I_I116V_F139L	0.248	23.6	−98	−85	15	68.5
	25A	M78I_I80F_L103I_I116V_F139L	0.080	19.4	−99	−84	16	73.5
	26A	L35F_M78F_I80G_I116V_F139W	0.240	19.3	−98	−60	164	45.5
								
		*cis*‐butene oxide (**2 a**)						
LEH‐P						−2	0.23 U mg^−1^	
Designs for (*R*,*R*)‐2 b	47B	M32L_L35M_M78I_I80L_V83L_I116M_F134Y	24.1	0.176	99	57	3	56.5
	48A^e^	M32L_L35G_M78L_I80W_L103I_F139L	9.00	0.152	97	24	60	56.0
	45A	M32L_L74I_L103V_L114W_I116L_F134G_F139W	5.72	0.112	96	18	1	–
	49A	L103V_L114W_I116L_F134G_F139W	4.14	0.048	98	31	6	59.0
	50A	M32L_L103V_F134Y_F139M	2.90	0.040	97	−3	15	73.0
Designs for (*S*,*S*)‐2 b	30A	L74W_I80F_L103I_I116V_F139L	0.496	25.2	−96	−73	11	70.5
	31A	L35F_M78F_I80A_I116V_F139W	0.320	23.7	−97	−41	100	56.0
	32A	L35W_L74F_I80G_I116V_F139L	0.080	21.8	−99	−82	15	57.5
	33B	M32L_I80W_L103I_F139L	0.168	21.0	−98	6	1	78.0
	26A	L35F_M78F_I80G_I116V_F139W	0.080	20.4	−99	−60	87	45.5
								
		*cis*‐stilbene oxide (**3 a**)						
LEH‐P						92	0.35 U mg^−1^	
Designs for (*R*,*R*)‐3 b	51A^e^	M32L_L35G_I80W_L103V_F139W	28.2	0.104	99	91	51	59.5
	52A	M32L_L35M_M78I_L103I_L114M_I116F	24.9	0.144	99	−97	22	61.0
	60A^e^	M32L_L35G_I80W_L103V_F139L	22.7	0.048	100	>99	52	65.0
	61B	M32A_I80V_L103V_L114W_I116V_F134G_F139L	22.1	0.136	99	92	3	66.5
	38A	M32L_M78L_I80V_L103V_F134W_F139L	22.0	0.352	97	>99	11	75.5
Designs for (*S*,*S*)‐3 b	62A	M32L_I80V_L103V_L114W_I116A_F134G_F139L	0.016	15.5	−100	–	<1	71.0
	63B	M78I_I80L_L103V_L114W_I116V_F134G_F139L	0.240	15.4	−97	40	7	67.0
	64C	M32A_L103V_L114W_I116A_F134G_F139L	0.152	15.3	−98	–	<1	57.5
	41B	M32L_L35M_L103I_L114M_I116F_F139L	0.072	15.0	−99	−95	21	62.0
	65B	L74I_M78F_L103V_L114A_I116V_F134W_F139M	0.008	14.7	−100	−28	2	66.0

[a] Mutations at the peripheral (*proRR* side) of the substrate binding pocket are underlined. Other mutations line the center (*proSS* side) of the substrate binding pocket. [b] Positive numbers: (*R*,*R*)‐diol preference; negative numbers: (*S*,*S*)‐diol preference. [c] Relative activities expressed in percentage of the activity with the template enzyme (indicated). Data from duplicate measurements with the same enzyme batch. [d] –, no activity. [e] Variants designed by Rosetta to exhibit (*S*,*S*)‐product selectivity but predicted by HTMI‐MD and found experimentally to produce (*R*,*R*)‐diol **3 b**.

After sequence verification genes were expressed in *E. coli* Top10 or 10β for enzyme production. All mutants were well expressed and could be isolated by His‐tag metal affinity chromatography. Typical yields were 50–150 mg per liter of culture. The redesigned LEHs were very stable and could be stored for over 2 years at −80 °C without loss of activity. Most variants showed a somewhat lower apparent melting temperature but more stable variants were also found (Table 2). Overall thermostability was well maintained with an average *T*_m,app_ of the redesigned enzymes of 62.7 °C as compared with 70 °C for the template LEH‐P and 50 °C for the wild‐type LEH. Activity assays were done by mixing purified enzyme with epoxide and measuring diol formation. Of the obtained 28 designs, 26 showed catalytic activity on the substrate they were designed for. Of these active variants, 66 % have an activity that is between 10–164 % of the wild‐type activity for their respective substrate.

Chiral analysis of the formed diols revealed that 21 (77 %) of the designed active variants showed the enantioselectivity predicted by MD simulations (10 % *ee* cutoff, Table 2). The cyclopentene oxide (**1 a**) designs were generated to examine if the design and selection protocol gave similar results to an earlier study in which 34 LEH variants were tested for the same substrate.^32^ Of the 10 new cyclopentene oxide designs, nine were active and had the predicted enantioselectivity. The inactive design was 45A, which was also selected as a design for butene oxide **2 a**, for which it did have a low catalytic activity. The five *proSS* designs produced (*S*,*S*)‐diol **1 b** with *ee* values of 56–85 %, while lower *ee* values were obtained with *proRR* designs for (*R*,*R*)‐**1 b**. The higher enantioselectivity of *proSS* designs is in agreement with previous observations.^32^

The butene oxide designs were generated to test the possibility to control regioselectivity of water attack with a very small prochiral epoxide substrate. All 10 variants were active and eight of them had the predicted enantioselectivity. As with cyclopentene oxide, the *proSS* designs performed better than the *proRR* designs, with the best mutant (32A) providing (*S*,*S*)‐diol **2 b** with 77 % *ee* The wrong predicted designs had very low enantioselectivity (*ee* of 2–6 %). Thus, for both of the small substrates the MD simulations were an effective tool for predicting LEH enantioselectivity.

For the largest substrate, stilbene oxide (**3 a**), six of the eight active variants showed the predicted enantioselectivity. This included two variants (51A, 60A) that were originally designed using Rosetta to have (*S*,*S*)‐**3 b** selectivity but for which the HTMI‐MD screening predicted preferential formation of (*R*,*R*)‐diol, which was in agreement with what was observed experimentally. In these cases, MD corrected the Rosetta design prediction. On the other hand, the combination of Rosetta and MD for design and prediction of enantioselectivity still gave two mismatches between prediction and experiment in case of **3 a** designs. Of these, variant 52A was exceptional because it was predicted to give (*R*,*R*)‐diol **3 b** whereas experimentally it produced (*S*,*S*)‐diol with high *ee* (97 %).

Despite the two prediction errors, the results show that highly enantioselective variants could also be designed computationally for stilbene oxide. The thermostable template enzyme had 92 % (*R*,*R*)‐diol selectivity, and four of the eight active new variants also displayed very high (*R*,*R*)‐preference (>91 % *ee*) whereas two other designed variants displayed high (*S*,*S*)‐diol preference (>91 % *ee*). The more extensive protein–substrate interactions with a bulky substrate likely result in more restricted reactive conformations of enzyme–substrate complexes, accompanied by high product enantioselectivity. Of the experimentally characterized variants for all three substrates, three have a relatively large (>100 Å^3^) increase in volume of active site, as calculated from the decreased side chain volume of introduced amino acids, and indeed these three variants have low or no catalytic activity (see Supporting Information). When variants with a predicted increase of the active site volume of >100 Å^3^ would have been removed from the libraries, only the primary Rosetta libraries for *cis*‐stilbene oxide would have shrunken (elimination of 178 of the 442 variants listed in Table 1).

**Catalytic properties of the best variants**. To examine how the use of Rosetta for redesign of LEH toward production of a specific diol enantiomer influences catalytic activity, we examined the kinetic properties (*k*_cat_ and *K*_M_) of the best mutants (Table 3). The LEH variants were produced using 1L cultures, giving again 50–150 mg purified protein per liter of broth, which was similar to the yield of the parent thermostable enzyme. Variants RR8 and SS16 were selected earlier as the best variants from a set of 37 designs for cyclopentene oxide **1 a** 32 and were included for comparison (Table 3). The results showed that 46C and RR8 had catalytic rate constants (*k*_cat_) for cyclopentene oxide that were similar to that of the thermostable template enzyme LEH‐P. Variants 43A and 24A had lower catalytic constants (2.5‐ and 7‐fold, respectively). Variant SS16 variant displayed an almost 2‐fold higher *k*_cat_ than the template LEH‐P. Thus, catalytic rates were quite well maintained. However, in most cases the *K*_M_ values were higher (8‐ to 76‐fold) for the redesigned LEHs. This might be due to an increase in the volume of the substrate binding cavity, which grew by 37 Å^3^ in mutant 46C that had a 76‐fold increase in *K*_M_ for **1 a**. The exception was 24A, which has a 1.4‐fold lower *K*_M_. In all designs for **1 a**, the specificity constant (*k*_cat_/*K*_M_) was decreased (Table 3). The kinetic measurements show that the main cause of the lower catalytic activities of most designs found during initial tests (Table 2) is due to a higher *K*_M_, not a lower *k*_cat_.

**Table 3:** Kinetic properties of computationally redesigned epoxide hydrolases.

Variant	Designed to produce	Assay substrate	*ee* [%]^[a]^	Preference	*k*_cat_ [s^−1^]^[b]^	*K*_M_ [mm] ^[b]^	*k*_cat_/*K*_M_ [M^−1^ s^−1^]
LEH‐P	–	**1 a**	13	(*R*,*R*)	0.035±0.004	4.2±0.2	7.9
RR8	(*R,R*)‐**1 b**	**1 a**	85	(*R*,*R*)	0.039±0.009	189±20	0.20
46C	(*R,R*)‐**1 b**	**1 a**	34	(*R*,*R*)	0.022±0.002	344±17	0.06
43A	(*S,S*)‐**1 b**	**1 a**	8	(*R*,*R*)	0.017±0.005^[c]^	35±4^[c]^	0.48
SS16	(*S,S*)‐**1 b**	**1 a**	−90	(*S*,*S*)	0.062±0.001	54±4	1.14
24A	(*S,S*)‐**1 b**	**1 a**	−85	(*S*,*S*)	0.005±0.001	3±0.2	1.9
LEH‐P	–	**2 a**	24	(*R*,*R*)	0.056±0.010	18±1	3.1
32A	(*S,S*)‐**2 b**	**2 a**	−82	(*S*,*S*)	0.063±0.002	225±9	0.28
LEH‐P	–	**3 a**	92	(*R*,*R*)	0.147±0.012	0.37±0.02	406
60A	(*R,R*)‐**3 b**	**3 a**	>99	(*R*,*R*)	0.052±0.006	0.06±0.01	890
52A	(*R,R*)‐**3 b**	**3 a**	−89	(*S*,*S*)	0.003±0.001	0.16±0.03	19
41B	(*S,S*)‐**3 b**	**3 a**	−94	(*S*,*S*)	0.002±0.001	0.19±0.02	10.5

[a] Calculated from multiple data points that is, product enantiomers in different reactions. [b] Averages of duplicate measurements with standard deviation. [c] Single measurement; margins from average coefficient of variation for **1 a** data.

The results for the variants designed to convert the other small substrate, *cis*‐butene oxide **2 a,** are similar. For this substrate, we examined 32A, which has the highest enantioselectivity for producing (*S*,*S*)‐diol and inverted enantioselectivity relative to the template. It showed an insignificant increase in *k*_cat_, but the *K*_M_ was much higher, resulting in a drop in *k*_cat_/*K*_M_ in comparison with the template LEH‐P.

The results were different for the variants designed to convert the bulky epoxide stilbene oxide **3 a** to (*R*,*R*)‐ or (*S*,*S*)‐diol. Here, *k*_cat_ values were lower than with wild‐type, whereas *K*_M_ values were better. The observation that *k*_cat_ values are lowered in the designs for **3 a** whereas NAC percentages during MD simulations (Table 2) were fine indicates that prediction of reactivities across different substrates using such short MD simulations is troublesome. This is not unexpected, as MD does not account for energy barriers along reaction coordinates.

The high enantioselectivities obtained for stilbene oxide variants, relative to the designs for the two other substrates, are likely due a more restricted conformational freedom in case of the bulky stilbene oxide. The better *K*_M_ values relative to those with small substrates might be due to the design procedure making the active site too spacious for small substrates, preventing a snug fit with good hydrophobic binding interactions. Stilbene oxide is also more bulky than the natural substrate limonene epoxide and the mutations created enough additional space (ca. 67 Å^3^ for mutant 60A, calculated from decreased side chain volumes) for tighter binding and a low *K*_M_. Consequently, the 3‐fold lower *k*_cat_ of mutant 60A with **3 a** was accompanied by a 6‐fold better *K*_M_, leading to an improved catalytic efficiency in 60A. Furthermore, the tight substrate binding caused the specificity constants (*k*_cat_/*K*_M_) to be higher for stilbene oxide **3 a** than for cyclopentene oxide **1 a** and *cis*‐2,3‐butene oxide **2 a** (Table 3). The improved *K*_M_ values for **3 a** relative to wild‐type were observed with all three tested stilbene oxide designs.

### Preparative scale conversions

The stilbene oxide enantioselectivities of designs 41B and 52A are higher than reported for other LEH variants tested on this substrate.^50^, ^51^, ^52^ To examine if these redesigned LEHs could be used in preparative scale conversions, the conversion of *cis*‐stilbene oxide **3 a** to the (*R,R*)‐ and (*S*,*S*)‐diols by variants 60A and 41B was examined under different reaction conditions, including varying temperatures and cosolvents (Table 4). Cosolvents were tested because the solubility of the substrates and products in water is low. Even in the presence of 10 % dioxane in 50 mm HEPES, pH 8.0, both *cis*‐stilbene oxide and the diols were only partially soluble when added at 50 mm. Under these conditions, the *cis*‐stilbene oxide remained visible as globular crystals while the (*R*,*R*)‐diol and the (*S*,*S*)‐diol formed needles. The LEH variants 41B and 60A were active in this suspension. Addition of cosolvents 1,4‐dioxane and THF and the presence of a biphasic system with a layer of ethyl acetate were studied. No conversion was observed with variant 60A in the biphasic system with ethyl acetate. Addition of 10 % dioxane gave the best conversion, yielding up to 78 % diol in 44 h at 30 °C. Remarkably, adding THF as co‐solvent (similar properties as 1,4‐dioxane) gave lower conversion than reaction conditions without cosolvent or with 10 % dioxane. The conversion of *cis*‐stilbene oxide by variant 60A was further improved (63 to 80 %) by increasing the reaction temperature from 30 to 40 °C. The best conditions (10 % dioxane and 40 °C) were combined and gave a conversion of 86 and 63 % for variants 60A and 41B, respectively. Increasing the dioxane concentration to 15 % was beneficial for the *proRR* variant 60A, yielding a conversion of 98 %, but drastically decreased conversion of **3 a** by the *proSS* variant 41B. The enantiomeric excess of the stilbene diol products was analyzed by chiral HPLC (Figure S2‐S4). For the best conversions the following results were obtained: >99 % *ee* for (*R*,*R*)‐stilbene diol (LEH 60A) and 88 % *ee* for the (*S*,*S*)‐stilbene diol (LEH 41B).

**Table 4:** Asymmetric synthesis of stilbene diols by computationally engineered enantiocomplementary epoxide hydrolases.^[a]^

	Temp [°C]	Time [h]	Conversion [%]	*ee* [%]
Production of (*R*,*R*)‐diol by LEH 60A				
HEPES 50 mm	30	44	63	–^[b]^
10 % 1,4‐dioxane	30	44	78	–
10 % THF	30	44	52	–
At 40 °C	40	44	80	–
10 % 1,4‐dioxane	40	24	76	–
10 % 1,4‐dioxane	40	48	86	–
15 % 1,4‐dioxane	40	48	98	>99 % (*R*,*R*)
Production of (*S*,*S*)‐diol by LEH 41B				
HEPES 50 mm	30	44	38	–
10 % 1,4‐dioxane	40	24	50	–
10 % 1,4‐dioxane	40	48	63	88 % (*S*,*S*)
15 % 1,4‐dioxane	40	48	18	–

[a] Reaction mixtures (total volume 1 mL) contained 10 mg *cis*‐stilbene oxide (final concentration 50 mm, suspension) and 6.37 mg enzyme (final concentration 320 μm) in 50 mm HEPES, pH 8.0. [b] –, not determined.

### Structural origin of enantioselectivity

**Figure 2:**
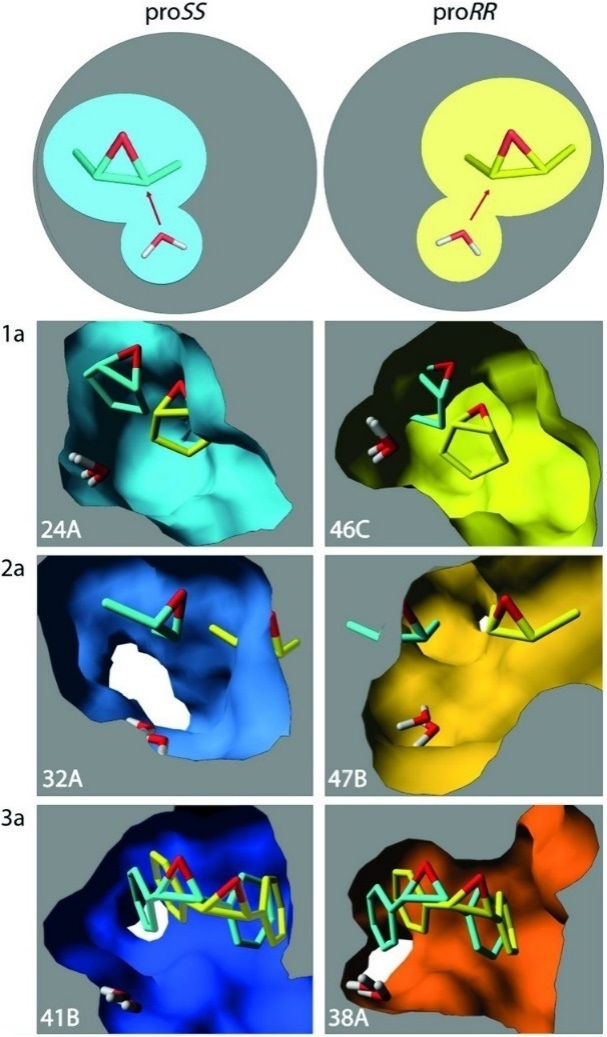
Structural basis of redesigned enantioselectivity. Shown are the active‐site cavities of three *proSS*‐ variants (blue shades, names indicated) and three *proRR* variants (yellow‐orange shades). The variants were designed for the substrates indicated at the left of each pair of panels. The reacting water molecules are shown. Hydrogen atoms of substrates are hidden for clarity. In each panel, both the designed enzyme with its substrate is shown, as well the position of the same substrate in the opposite design (substrates in *proSS* designs in cyan, substrates in *proRR* designs in yellow). This shows pronounced differences in substrate positioning and how steric hindrance steers product enantioselectivity.

To provide a structural explanation for the observed mutant enantioselectivities we inspected the Rosetta designed structures and the average HTMI‐MD structures of selected mutants. According to these models, the nucleophilic water molecule stays in virtually the same position (Figure 1, Figure 2). This agrees with the X‐ray structure, in which the catalytic water has an unusually low B‐factor^48^ indicating a precise orientation due to H‐bonds from Tyr53, Asn55, and Asp132. The enantioselectivity of the enzyme is therefore determined by the positioning of substrate relative to this water. In all of the modeled structures, the positional differences that influence enantioselectivity can globally be described as a sliding motion of the epoxide carbon atoms in front of the nucleophilic water molecule (Figure 2). Mutations causing the substrate to reside more toward the center of the dimeric enzyme (i.e., near β strands β4, β5, β6, and helix H4, see legend of Figure 1 for residue numbers) will lead to attack on the (*R*)‐configured carbon of the epoxide ring, resulting in an (*S*,*S*)‐diol. Vice versa, *proRR* attack will dominate if the substrate is positioned more toward the peripheral side (i.e., near β strand β3 and helices H1 and H3).

In the models of the *proSS*‐selective variants that convert substrates **1 a** and **2 a** with high enantioselectivity (*ee* >75 %), the dominant substrate orientations are achieved by steric hindrance introduced by mutations at the *proRR* side (e.g., L35W/F, L74W/F, M78F, and I80W/F). These mutations will promote positioning of the substrate more toward the central (*proSS*) side. At the same time, space‐creating mutations on the *proSS* side (I116V and F139L) will further increase the preference for (*S*,*S*)‐diol formation. Indeed, designs for substrate **1 a** and **2 a** carrying both L35W and I116V gave an (*S*,*S*)‐diol preference of >73 % *ee* (Table 2). Furthermore, also in mutant 32A the steric hindrance mutations L35F and L74F on the peripheral (*proRR*) side are accompanied by I116V, leading to (*S*,*S*)‐butanediol formation with the best *ee* of 77 %.

The origin of the opposite (*R*,*R*)‐selectivity of variants with substrates **1 a** and **2 a** can be explained in a similar way. It is likely that increased steric hindrance due to mutations on the central side of the substrate binding cavity (e.g., mutations L114W, I116F/M) encourage positioning of the substrate closer to the peripheral (*proRR*) side of the binding pocket. This is visible in mutant 46C for substrate **1 a** (e.g., mutation I116F) and possibly in 47B for substrate **2 a** (e.g., mutation I116M). However, such a steric effect is not clear in all predicted *proRR* mutant structures, and some designs indeed show low enantioselectivity (e.g., 45A, 50A). The weakly (*R*,*R*)‐diol selective variant 63B contains mutation I116V, creating space on the central (*proSS*) side, but it is accompanied by L114W, reducing that space. The combined effect of such mutations appears difficult to rationalize in view of effects of side chain interactions and dynamics.

The mutants designed for stilbene oxide **3 a** have high product enantioselectivities but the mutations that cause them appear to not only involve steric effects. The majority of the **3 a** designs showed (*R,R*)‐preference, including variant 63B designed for (*S*,*S*)‐enantioselectivity. Surprisingly, the two most (*S*,*S*)‐diol selective mutants for **3 a** (52A, 41B) carried mutation I116F, a mutation that introduces steric hindrance at the central side and in case of substrates **1 a** and **2 a** favors (*R*,*R*)‐selectivity (see above). The unexpected (*S*,*S*)‐diol preference might be due to π–π interactions between the substrate and the newly introduced aromatic ring of Phe116. The same selectivity by attraction might hold for one of the best (*R*,*R*)‐selective mutants: variant 60A (*ee* >99 %) carries the I80W mutation at the peripheral (*proRR*) side and no steric hindrance introducing mutation on the *proSS* site. For the other strongly (*R*,*R*)‐selective mutant, 38A with *ee* >99 %, the introduction of steric hindrance at the *proSS* side (F134W) can explain the improvement in enantioselectivity along the same lines as for substrates **1 a** and **2 a**. The wild‐type (*ee* >90 % (*R*,*R*)‐diol) also has aromatic functionality with Phe134 at the peripheral (*proRR*) region. Thus, it appears possible that enantiomeric preference with stilbene oxide is partially determined by an influence of attractive π–π interactions on binding orientations of the substrate.

Instead of a translational shift in the position of substrate, the stereoselectivity of LEH variants could also be influenced by rotating the substrate in the binding pocket by 180° along an axis formed by the epoxide oxygen and the spot in between the two epoxide carbon atoms. This would switch the orientation of the epoxide carbon atoms relative to the nucleophilic water. However, such substrate rotations were not observed in any of the models or during MD simulations. Also attempts by us to dock substrates into productive orientations featuring such a rotation were unsuccessful.

## Discussion

Changing enantioselectivity of limonene epoxide hydrolase by directed evolution has been extensively investigated by Reetz, Sun and co‐workers, focusing on improving directed evolution strategies including optimizing target positions and positional diversity in libraries.^47^, ^50^, ^52^ Such well‐optimized directed evolution protocols still rely on substantial experimental screening by chiral chromatography, which triggered us to examine if computational methods could be developed to enrich libraries and replace most of the laboratory screening by computational screening of protein libraries.^60^ In silico screening methods have been used earlier to design cocaine hydrolyzing enzymes with improved catalytic efficiency,^61^, ^62^ but also to design libraries of a cytochrome P450 harboring variants with controlled selectivity,^63^ and to increase amidase activity in an esterase.^64^

The results show that design of small sets of mutants with Rosetta and screening by MD simulations could indeed generate LEH variants with desired enantioselectivity. Rosetta design targeted 11 positions at the same time, with a nine‐residue diversity per position. MD screening was done by multiple ultra‐short simulations with on‐the‐fly scoring of reactive conformations and allowed to screen thousands of Rosetta designs. This CASCO protocol^32^ decreased the size of the library required, and for each substrate only five variants per desired enantiomer were used to find mutants with enhanced enantioselectivity. In case of cyclopentene oxide **1 a**, directed evolution^47^, ^50^, ^51^, ^52^ and previous computational design^32^ gave (*S*,*S*)‐selective LEH variants producing the diol with similar *ee* as found here (60–95 % *ee*, Table 2).

A comparison of predicted enantioselectivities as calculated from NAC percentages (Table 2) shows that there is good overall agreement only in qualitative terms, that is, (*R*,*R*)‐ or (*S*,*S*)‐diol stereopreference was correctly predicted for 77 % of the variants using multiple short MD simulations with independent initialization. On the other hand, within a set of five designs, experimental activities for individual variants did not correlate with their computed NAC%, showing that the MD simulations as performed here do not provide quantitative information on catalytic rates. Note, however, that rates shown in Table 2 are strongly influenced by both *K*_M_ values and do not reflect *k*_cat_. Furthermore, in a broader sense, for each of the three substrates examined, the set of designs that gave the highest NAC% in MD also showed the highest average activity. Thus, on average the *proRR* designs for **3 a** were on average more active than its *proSS* designs and also gave the highest NAC percentages for **3 a**. For the other two substrates *proSS* designs were more active and gave higher NAC%.

In this study, the best enantioselectivities were clearly obtained with stilbene oxide **3 a**. Whereas most designs produced (*R*,*R*)**‐**diol **3 b** (with *ee* >99 % for two variants), enantiocomplementary mutants yielding (*S*,*S*)‐**3 b** were also found, with a highest *ee* of 97 %. With butene oxide **1 a**;^51^, ^52^ the obtained variants showed high and modest enantioselectivity in the production of (*R*,*R*)‐ and (*S*,*S*)‐diols, respectively. The LEH mutants found for stilbene oxide could be used to produce enantiopure (*R*,*R*)‐diol and (*S*,*S*)‐diol at preparative scale, indicating the potential of this approach to generate a practically useful biocatalyst. Ring opening of stilbene oxide was tested earlier using mutants optimized on cyclopentene oxide and cyclohexene oxide, which resulted in highly (*R*,*R*)‐selective variants (with *ee* 99 %) but only modest (*S*,*S*)‐selective variants (*ee* 44 %).^65^ The results suggest that the likelihood of obtaining high enantioselectivity is much better with the bulkier stilbene oxide than with the smaller substrates, with the two phenyl rings of stilbene oxide offering more opportunities for steric hindrance as well as van der Waals and π–π interactions.

Similar to what we found with redesigned aspartases catalyzing asymmetric hydroamination of acrylates,^33^ we observed that the lower activity found with most designs for **1 a** and **2 a** as compared with the template (Table 2) was not due to decreased *k_cat_*, but due to an increase in *K*_M_ (Table 3). From a practical point of view, this is less disturbing than the opposite, because for reasons of process economy preparative‐scale applications must be carried out at high substrate concentrations anyway. Recently, Sun et al.^65^ attributed low activity of LEH variants obtained by directed evolution to misalignment of the nucleophilic water, epoxide carbon and epoxide oxygen for an S_N_2 reaction, caused by increased flexibility in the active site, but without reporting kinetic data. This explanation likely does not hold for the computationally redesigned enzyme studied by us because such a misalignment would decrease *k_cat_* and probably also *K*_M_, which is not what we observe, except for some stilbene oxide designs (Table 3). Proper alignment of the nucleophilic water and reacting substrate atoms for S_N_2 is one of the constraints in the Rosetta design process and a NAC criterion during MD screening.

Surprisingly, four of the observed enantioselectivities with stilbene oxide **3 a** were opposite to the Rosetta design target (Table 2). Two of these were corrected in MD simulations. To understand how these incorrect designs could emerge, we examined sequences and structures of Rosetta‐optimized enzyme–substrate complexes of all substrates (see above). For substrates **1 a** and **2 a**, the observed enantioselectivities could be explained by the combination of steric hindrance and space‐creating mutations from the peripheral and central side of the substrate binding pocket, acting together to steer the substrate in a *proRR* or *proSS* binding mode. This did not hold for several of the mutants designed for stilbene oxide **3 a**. For both variants that are strongly *proSS* selective for **3 a** the only bulk introducing mutation is I116F, which would be expected to introduce steric hindrance at the central side of the cavity and thus stimulate *proRR* selectivity. Also, for one of the two most *proRR* selective variants (60A, *ee* >99 %) the I80W mutation would be expected to decrease space at the peripheral side and thereby stimulate *proSS* selectivity. Thus, it appears that effects of mutations on bulkiness are poorly related to stereopreference in case of aromatic substrate **3 a**, suggesting that electronic effects that are not well modeled, such as π–π interactions, dominate over steric factors in determining substrate positioning or reactivity.

Standard computational design and MD simulations do not explicitly account for π–π interactions to save computation time. MD simulations can give more realistic results in cases where aromatic interactions play a role when the force field is adapted with an additional noncovalent interaction term.^66^ Whether the main effect of π–π interactions is on binding, conformational dynamics, or reactivity of bound substrates is unclear at present. Recently, Zaugg et al.^67^ investigated the origin of enantioselectivity of *Aspergillus niger* epoxide hydrolase in the conversion of the chiral substrate phenyl glycidyl ether (PGE). Molecular dynamics simulations suggested that the protein does not differentiate enantiomers based on binding mode, and free energy calculations did not show significant differences between (*R*)‐ and (*S*)‐PGE binding either. The authors suggested that the enantioselectivity is due to kinetic differences. For such an α/β‐hydrolase fold epoxide hydrolase a computational analysis is more complicated due to the multiplicity of reaction pathways and chemical steps. Earlier, Lau et al.^68^ studied murine epoxide hydrolase with (1*S*,2*S*)‐*trans*‐2‐methylstyrene oxide using ab initio and density functional calculations, and suggested the importance of interactions between the substrate's phenyl group and aromatic residues in the binding pocket. Moreover, Lind and Himo^69^ published the reaction mechanism of a soluble epoxide hydrolase (StEH1) converting styrene oxide. They proposed coplanarity of the oxirane C1‐C2 carbons with the substrate's phenyl substituent, and π–π interactions between this phenyl group and a histidine and phenylalanine to be important for the stabilization of the transition state and for the selectivity of the enzyme. Rinaldi et al.^70^ proposed that substrate‐dependent LEH regioselectivity is related to reorganization of the active site toward each ligand. Based on QM/MM calculations, they confirmed that substrate‐specific LEH regioselectivity is due to both conformational and electronic parameters.

### Conclusions

We conclude that computational design and MD simulations are well able to predict and screen enantioselectivity of LEH variants in case of small aliphatic substrates. Whereas highly selective variants for production of aromatic diols can be obtained, prediction accuracy is lower. In view of the effect of interactions involving aromatic groups on epoxide hydrolase enantioselectivity, rapid scoring methods that more accurately include effects of π–π interactions appear necessary to further improve computational screening of LEH variants acting on aromatic substrates.

## Experimental Section

**Materials**. The *meso*‐epoxides and their corresponding diols, oligonucleotides for mutagenesis, organic solvents and glycerol were purchased from Sigma–Aldrich. Restriction enzymes and PfuUltra Hotstart PCR Master Mix were obtained from New England Biolabs and Agilent, respectively. Ni‐NTA resin was purchased from GE Healthcare Life Sciences. SYPRO orange was obtained from ThermoFisher Scientific. Complete protease inhibitor cocktail tablets were bought from Roche. Media components were obtained from Difco (BD Biosciences).

**Computational design**. To design LEH variants for production of highly enantioenriched diols from *meso*‐epoxides the previously developed CASCO strategy was used with only minor modifications.^32^ The X‐ray structure of the wild‐type LEH (Protein Databank 1NWW) was used for computational design. Eleven positions around the active site (M32, L35, L74, M78, I80, V83, L103, L114, I116, F134 and F139) were selected to mutate simultaneously to any of the nine hydrophobic residues (AFGILMPVW). Each of the three substrates was docked in the enzyme active site, either in a *proRR* or *proSS* conformation using Rosetta enzyme design^34^, ^71^ Catalytically productive binding modes were defined using a constraint file as previously.^32^ This geometric description of how the substrate should be bound included the obligation to form H‐bonds between the epoxide oxygen and D101 and between the nucleophilic water and D132, Y53, and N55. Furthermore, the water oxygen had to be close (1.8 Å) to the attacked carbon atom while the angle of nucleophilic attack (i.e., from water oxygen, attacked carbon, and epoxide oxygen) should be close to 180°. Another constraint was that the distance between the nucleophilic water and the non‐attacked epoxide carbon atom should be >3.8 Å. To hinder undesired substrate‐binding orientations, a bulky residue (W, F or Y) was introduced at one of the eleven target positions, as this may reduce binding poses not contributing to the desired selectivity.^32^ Rosetta enzyme design was used to simultaneously mutate the remaining ten residues to any of the nine hydrophobic residues and sequence‐conformational space was searched for substrate‐bound structures with low energy and a catalytically productive binding mode.

High‐throughput‐multiple independent MD simulations (HTMI‐MD) were used for in silico screening of the generated libraries and to rank the primary designs with an orthogonal tool (Wijma et al., 2014). Independent initialization of multiple trajectories increases the conformational space sampled by molecular dynamics and decreases the computational cost of the screening step relative to a single long MD run.^46^ The reactivity and selectivity of each mutant were predicted by scoring the fraction of snapshots in which the enzyme–substrate complex is in a *proRR* or *proSS* near‐attack conformation (NAC). The latter are defined by geometric constraints (Figure 1), which should be fulfilled for a reaction to become feasible. The geometric criteria for *proRR* and *proSS* attack conformations were as defined using published quantum mechanical modeling.^72^ The ratio of *proRR* and *proSS* NAC frequencies were considered to reflect regioselectivity of attack and thus product enantioselectivity according to Equation (1),

(1) $$e.e.\;=\frac{\left({\left[NAC\right]}^{proRR}\;-{\left[NAC\right]pro}^{SS}\right)}{\left({\left[NAC\right]}^{proRR}+{\left[NAC\right]}^{proSS}\right)}$$

in which *ee* is the predicted enantiomeric excess, [NAC]^proRR^ and [NAC]^proSS^ indicate the fraction of snapshots in which the enzyme–substrate complex is in a *proRR* or *proSS* conformation, respectively. Positive values indicate predicted (*R*,*R*)‐diol preference, negative values (*S*,*S*) selectivity.

The only modification from the existing procedures are listed in this paragraph. More design calculations were done than previously, and also more seeds per MD simulation.^32^ For the current study, approximately 25 thousand design calculations were run per target substrate (Table 1), which is two times more than previously. Like earlier,^32^ a stepwise scheme to rank the variants was adopted in which variants were eliminated as soon as they failed a criterion (Table 1). The selection criteria were based on *ee* ^pred^ and [NAC] values for the preferred enantiomer. The criteria differed per target substrate and are listed in Table 1. For each designed new variant 20–80 independently started MD simulations of 10 ps were used (previously maximally 20 MD simulations). For the final variants also five MD simulations of 100 ps were performed.

Finally, the best ranked mutants were visually inspected. For each of the targeted product enantiomers, only variants predicted to have a high enantioselectivity were visually inspected, starting with those variants that were predicted to have the highest fractions of NACs. The main reasons for elimination of designs were a too spacious active site cavity or an orientation of the substrate relative to the water that seemed in disagreement with the predicted enantioselectivity (Table S1). Only 15 of the 45 inspected designs were eliminated at the stage of visual inspection. Furthermore, no mutations were added at this stage even though this is common in the field.^73^ As a result, the visual inspection only took a few hours.

**Mutagenesis, expression and purification**. For the expression of LEH and variants thereof in *E. coli* a pBAD based expression vector was used. This vector contained the gene of the thermostable variant LEH‐P with an N‐terminal hexa‐histidine tag.^56^ The computationally designed variants of LEH‐P were constructed by QuikChange site‐directed mutagenesis using *Pfu* Ultra Hotstart PCR Mastermix (Agilent), combining multiple mutations in a single primer when possible, and omitting sequence verification between individual mutation steps. PCR reactions, transformations, plating and final sequencing were done in microtiter plate format.^74^ The obtained plasmids were used to transform chemically competent *E. coli* Top10 or *E. coli* NEB10β cells (Thermo Fischer Scientific). For expression, cells were grown overnight in 5 mL Luria‐Bertani broth at 37 °C. All cultures were supplemented with 50 μg mL^−1^ ampicillin. The resulting culture was used to inoculate 500 mL Terrific Broth medium and incubated at 37 °C and 135 rpm. When an OD_600_ of 0.6 was reached, expression was induced by adding 0.04 % (*w*/*v*) arabinose and growth was continued at 30 °C and 135 rpm. After 24 h the cells were harvested by centrifugation at 6700 *g* and 4 °C for 15 min.

For protein isolation, cells were resuspended in 50 mm HEPES buffer, pH 8, containing 500 mm NaCl (3 mL per gram of cells) and half of a protease inhibitor cocktail tablet to prevent proteolysis (Roche Applied Science). After sonication (60×10 s with 20 s intervals, Labsonic M), the extract was centrifuged at 35 200 *g* and 4 °C for 1 h. The supernatant was collected and the enzyme of interest was purified by gravity‐flow affinity chromatography under native conditions using Ni‐NTA agarose resin (Thermo Fischer Scientific). The protein concentration of the collected fractions was determined by a Bradford assay, and selected fractions were desalted by Econo‐Pac 10DG desalting columns (Bio‐Rad). The purity of the prepared enzymes (yield 50–150 mg per L TB medium) was analyzed by SDS‐PAGE. Enzymes were stored at −80 °C until further use.

**Catalytic properties**. Chiral chromatography was used to determine the enantioselective hydrolysis of three *meso*‐epoxides (*cis*‐2,3‐butene oxide, cyclopentene oxide and *cis*‐stilbene oxide) to chiral diols. In case of *cis*‐2,3‐butene oxide and *cis*‐stilbene oxide, 5 mg of purified enzyme was added to 50 mm substrate (virtual concentration of the suspension) in 50 mm HEPES pH 8 (800 μL total reaction volume). After incubation of the reaction mixture at 30 °C for 1 h, 500 μL of 5 m NaCl was added and the samples were extracted three times with 600 μL ethyl acetate. The combined extracts containing diols were dried by adding anhydrous sodium sulfate, concentrated under vacuum, and dissolved in 100 μL ethyl acetate. For cyclopentene oxide, reactions were done in a similar way after which 250 mg K_2_CO_3_ was added to the reaction mixture followed by extraction for two times by 600 μL of n‐butanol. The combined extract was dried, concentrated under vacuum and resuspended in 100 μL n‐butanol.

In case of *cis*‐2,3‐butene oxide and cyclopentene oxide, chiral analysis was carried out by injecting 2 μL of the extracts into an Agilent gas chromatograph equipped with a flame ionization detector and a Hydrodex β‐TBDAc column (Aurora Borealis, initial temperature 40 °C, 10 °C min^−1^ to 150 °C, hold 20 min). For *cis*‐stilbene oxide and its diols, samples were analyzed by HPLC on a Luxcellulose‐3 column (Phenomenex, Utrecht, the Netherlands) with heptane/2‐propanol (90/10) as the mobile phase (flow rate 1 mL min^−1^, detection at 254 nm). Samples from preparative scale reactions with *cis*‐stilbene oxide and the diols were also analyzed by HPLC on a Chiralpak AS‐H column (Daicel Corp, Illkirch, France) with n‐hexane/2‐propanol (90:10 (*v*/*v*) as the mobile phase (1 mL min^−1^, detection at 254 nm). Enantiomeric excess (*ee*) values were calculated from concentrations of the (*R*,*R*)‐ and (*S*,*S*)‐product enantiomers.

To obtain steady‐state kinetic parameters, initial velocities at different substrate concentrations were determined and fitted with the Michaelis–Menten equation.

**Determination of the apparent melting temperature**. The ThermoFluor assay was used to determine the apparent melting temperatures ($T_{m}^{app}$ ) of the purified enzyme variants.^75^ This method is based on monitoring the change in fluorescence of Sypro Orange dye during the thermal unfolding of a protein. The dye binds to the unfolded and exposed hydrophobic protein core, increasing its fluorescence signal. The assays were done as described before.^26^

**Synthesis of stilbene diols**. Reaction mixtures (total volume 1 mL) contained 10 mg of *cis*‐stilbene oxide (final concentration 50 mm, suspension) and 6.37 mg of enzyme (final concentration 320 μm) in 50 mm HEPES, pH 8.0, and were incubated (at 30 or 40 °C, 135 rpm) for 48 h. Substrate and products were extracted three times by 4 mL ethyl acetate, dried over MgSO_4_ and filtered. The solvent was removed by a rotary evaporator. The residue was analyzed by ^1^H NMR for conversion and by chiral HPLC to determine the enantiomeric excess. Chiral HPLC was used as described above.

*H.J.W. designed the mutants; H.A. constructed, isolated, and characterized the mutants; H.A., P.J., and D.I.C. measured catalytic activities; H.J.W., H.A., and E.B. interpreted the mutants; D.I.C. and M.T. performed preparative experiments; H.A., H.J.W., E.B., M.T., and D.B.J. wrote the paper; H.J.W. and D.B.J. supervised the work*.

## Conflict of interest

*The authors declare no conflict of interest*.

## Supporting information

As a service to our authors and readers, this journal provides supporting information supplied by the authors. Such materials are peer reviewed and may be re‐organized for online delivery, but are not copy‐edited or typeset. Technical support issues arising from supporting information (other than missing files) should be addressed to the authors.

Supplementary
