# Supplementary material for: Computational Design of Enantiocomplementary Epoxide Hydrolases for Asymmetric Synthesis of Aliphatic and Aromatic Diols
Source: Chembiochem. 2020 Mar 5;21(13):1893–904. doi: 10.1002/cbic.201900726 (PMC7383614; doi:10.1002/cbic.201900726)
Supplement: Supplementary file 1 — Supplementary [file CBIC-21-1893-s001.pdf]

CHEMBIOCHEM

Supporting Information

Computational Design of Enantiocomplementary Epoxide Hydrolases for Asymmetric Synthesis of Aliphatic and Aromatic Diols

Hesam Arabnejad, Elvira Bombino, Dana I. Colpa, Peter A. Jekel, Milos Trajkovic, Hein J. Wijma, and Dick B. Janssen*[a]

cbic_201900726_sm_miscellaneous_information.pdf

Author Contributions

H.J.W. designed the mutants; H.A. constructed, isolated, and characterized the mutants; H.A., P.J., and D.I.C. measured catalytic activities; H.J.W., H.A., and E.B. interpreted the mutants; D.I.C. and M.T. performed preparative experiments; H.A., H.J.W., E.B., M.T., and D.B.J. wrote the paper; H.J.W. and D.B.J. supervised the work.

Supporting information

Table S1. Visual inspection results.

Substrate	**1a**		**2a**		**3a**	
Designed preference	*proRR*	*proSS*	*proRR*	*proSS*	*proRR*	*proSS*
**Visually inspected designs**	**7**	**7**	**5**	**6**	**12**	**8**
Rejected during visual inspection because:						
· mutant structure seemed unusually flexible	1					
· active site seemed too spacious; repositioning of the substrate was likely	1	1		1	1	3
· mutated side chain making hydrogen bonds to the epoxide oxygen, suggesting design inactivity		1				
· water positioned in such a way that attack on the (NAC predicted) non-preferred carbon atom seemed feasible					6	
**Totally rejected**	**2**	**2**	**0**	**1**	**7**	**3**
Selected for experimental characterization	5	5	5	5	5	5

Visual inspection was carried out on averaged structures from the 5 × 100 ps MD simulations.

Table S2. Changes in side chain volumes of the mutants

Name variant	mutations	Change in side chain volume[A] (A^3)
1A	M32L_M78W_I80V_L103F_F139W	59
43A	L74I_L103V_F134Y_F139W	15
59A	M78L_L103V_L114G_I116F_F139I	-95
45A	M32L_L74I_L103V_L114W_I116L_F134G_F139W	-39
46C	M32L_L103V_L114A_I116F_F139W	-37
3A	M32L_L35W_I80A_I116V_F139W	-9
4C	M32L_L35W_I80G_V83I_I116V_F139W	-9
24A	M32A_M78I_I80F_L103I_I116V_F139L	-76
25A	M78I_I80F_L103I_I116V_F139L	-19
26A	L35F_M78F_I80G_I116V_F139W	-45
47B	M32L_L35M_M78I_I80L_V83L_I116M_F134Y	25
48A	M32L_L35G_M78L_I80W_L103I_F139L	-48
49A	L103V_L114W_I116L_F134G_F139W	-39
50A	M32L_L103V_F134Y_F139M	-24
30A	L74W_I80F_L103I_I116V_F139L	20
31A	L35F_M78F_I80A_I116V_F139W	-26
32A	L35W_L74F_I80G_I116V_F139L	-56
33B	M32L_I80W_L103I_F139L	28
26A	L35F_M78F_I80G_I116V_F139W	-45
51A	M32L_L35G_I80W_L103V_F139W	-28
52A	M32L_L35M_M78I_L103I_L114M_I116F	11
60A	M32L_L35G_I80W_L103V_F139L	-67
61B	M32A_I80V_L103V_L114W_I116V_F134G_F139L	-173
38A	M32L_M78L_I80V_L103V_F134W_F139L	-21
62A	M32L_I80V_L103V_L114W_I116A_F134G_F139L	-154
63B	M78I_I80L_L103V_L114W_I116V_F134G _F139L	-97
64C	M32A_L103V_L114W_I116A_F134G_F139L	-192
41B	M32L_L35M_L103I_L114M_I116F_F139L	0
65B	L74I_M78F_L103V_L114A_I116V_F134W_F139M	-67
RR8	M32L_L74I_I80V_L103F_F139L	-19
SS16	M32L_L35W_L74F_M78F_I80A_I116V_F139L	-26

Changes in side chain volume were calculated using the side chain volumes given in T.E. Creighton, Proteins: Structures and Molecular Properties Second Edition (1992) W.H. Freeman.

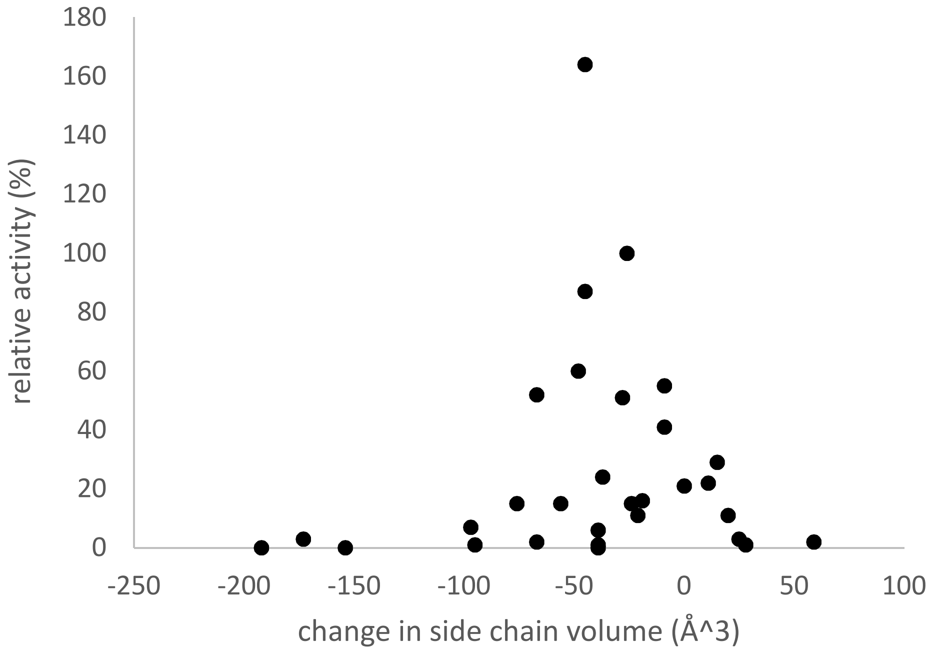

Figure S1. Side chain volume as a predictor for loss of catalytic activity. The relative activities are from Table 2 in the manuscript and the total changes in side chain volume are from Table S1. The results suggest both a large increase and a large decrease in active site volume are detrimental for activity.

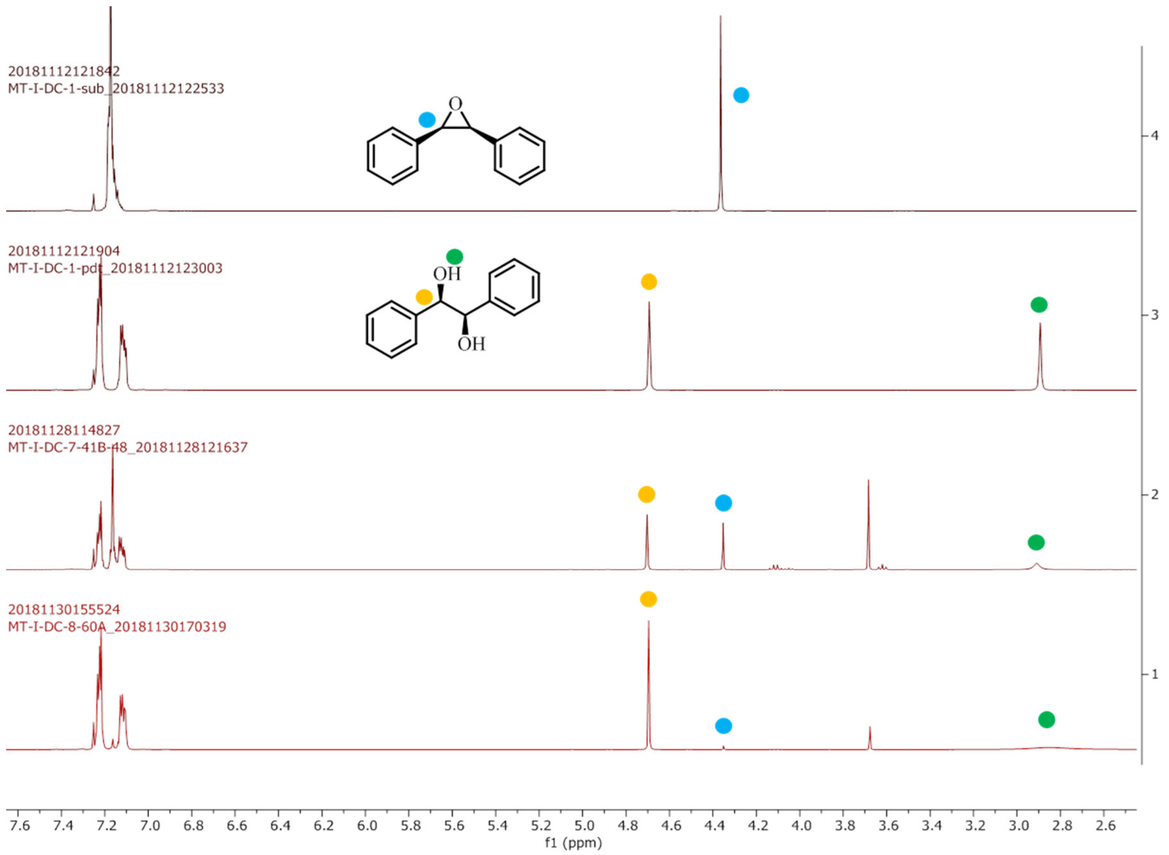

Figure S2: ^{1}H-NMR analysis of the conversion of *cis*-stilbene oxide to stilbene diols by LEH mutants 41B and 60A. From top to bottom: the substrate *cis*-stilbene oxide **3a**, product *R,R*-diol, the product after conversion of *cis*-stilbene oxide by LEH 41B (63% conversion, 88% *S,S*) and the product after conversion by LEH 60A (98% conversion, >99% *R,R*). The enantiomeric excess was analyzed by chiral HPLC.

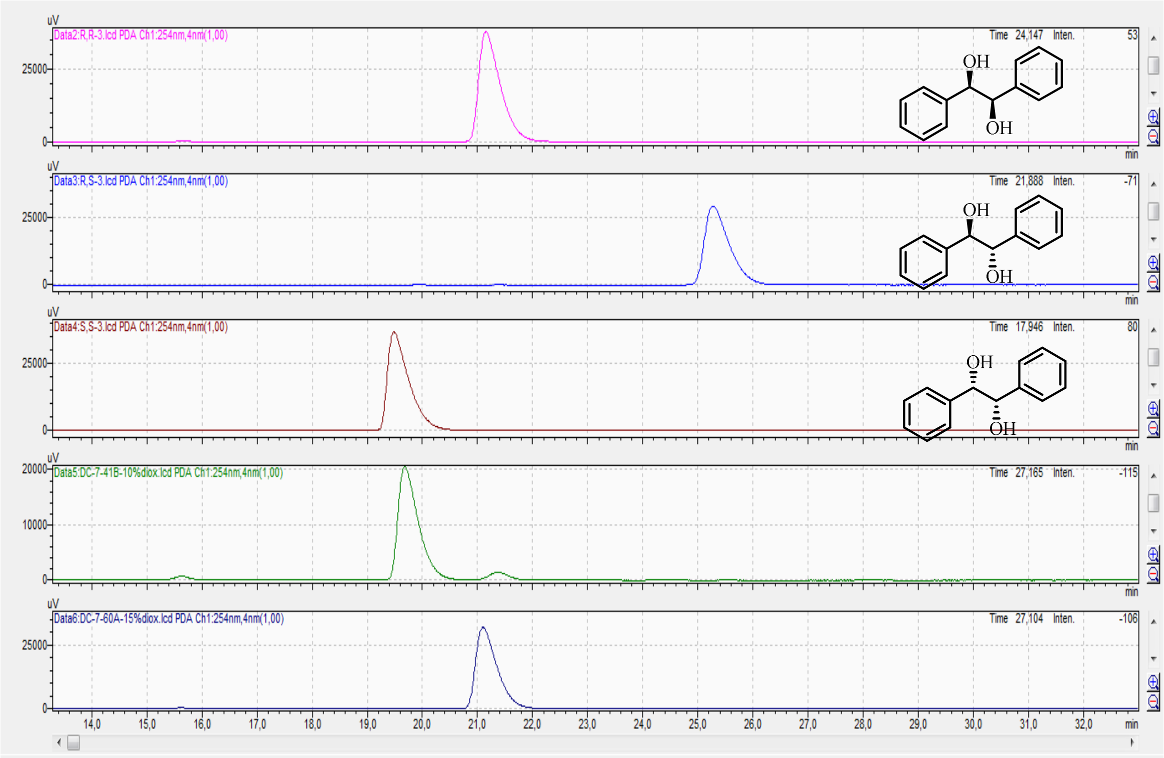

Figure S3: Chiral HPLC. From top to bottom: the substrate *cis*-stilbene oxide **3a**, product *R,R*-diol, the product after conversion of *cis*-stilbene oxide by LEH 41B and the product after conversion by LEH 60A.

<Sample Information>

Sample ID	:		
Data Filename	: DC-7-41B-10%diox.lcd		
Method Filename	: C3 90_10 45min fl 0.5.lcm		
Batch Filename	: 20181204.lcb		
Vial #	: 1-74	Sample Type	: Unknown
Injection Volume	: 5 uL		
Date Acquired	: 4-12-2018 18:00:40	Acquired by	: System Administrator
Date Processed	: 27-5-2019 13:56:07	Processed by	: System Administrator

<Chromatogram>

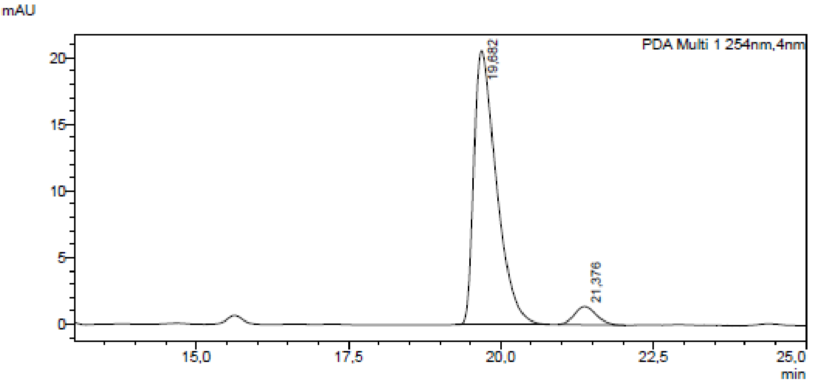

<Peak Table>

PDA Ch1 254nm

Peak#	Ret. Time	Area	Height	Conc.	Unit	Mark	Name
1	19,682	530275	20557	93,923			
2	21,376	34308	1377	6,077			
Total		564583	21934				

<Sample Information>

Sample ID	:		
Data Filename	: DC-7-60A-15%diox.lcd		
Method Filename	: C3 90_10 45min fl 0.5.lcm		
Batch Filename	: 20181204.lcb		
Vial #	: 1-75	Sample Type	: Unknown
Injection Volume	: 5 uL		
Date Acquired	: 4-12-2018 18:46:07	Acquired by	: System Administrator
Date Processed	: 27-5-2019 13:54:00	Processed by	: System Administrator

<Chromatogram>

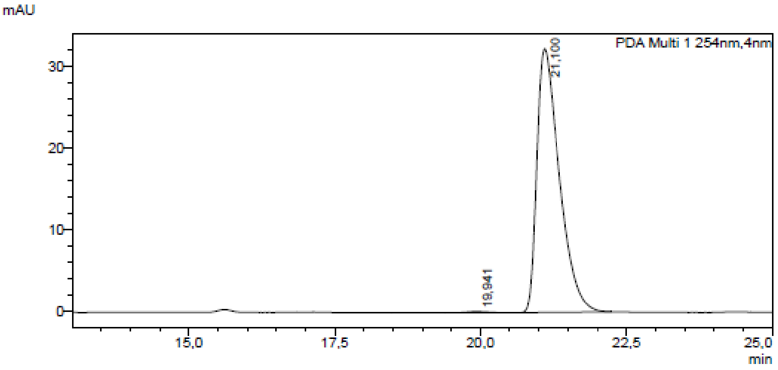

<Peak Table>

PDA Ch1 254nm

Peak#	Ret. Time	Area	Height	Conc.	Unit	Mark	Name
1	19,941	3016	138	0,348		M	
2	21,100	863303	32204	99,652			
Total		866319	32342				

Figure S4: HPLC analysis reports. Product after conversion of *cis*-stilbene oxide by LEH 41B and product after conversion by LEH 60A.
